# The Influence of Pre-Harvest Application of *Bacillus velezensis* LYB73 on the Rhizosphere Soil Properties, Microbial Communities, Fruit Quality, and Flavor Characteristics of Different Varieties of Peaches

**DOI:** 10.3390/foods15111852

**Published:** 2026-05-23

**Authors:** Chen Yang, Xinhui Wang, Chengxiong Kuang, Xiao Huang, Qiang Li, Dayu Liu, Yang Tao, Bingliang Liu

**Affiliations:** 1 College of Food and Biological Engineering, Chengdu University, Chengdu 610106, China; 2 Institute for Advanced Study, Chengdu University, Chengdu 610106, China; 3 Sichuan-Xizang Medicinal Resource Breeding and Standardization Team, Engineering Research Center of Sichuan-Xizang Traditional Medicinal Plant, Chengdu University, Chengdu 610106, China

**Keywords:** peach, *Bacillus velezensis*, rhizosphere microorganisms, fruit quality, variety specificity

## Abstract

The effects of pre-harvest application of *Bacillus velezensis* LYB73 on rhizosphere soil properties, microbial communities, fruit quality, and flavor-related traits in different peach cultivars are still not well understood. In this study, three peach cultivars, “Jinxia” (JX), “Wanhujing” (WHJ), and “Youpantao” (YPT), were subjected to *B. velezensis* LYB73 treatment or a sterile-water control under field conditions. Rhizosphere bacterial (16S rRNA) and fungal (ITS) communities were analyzed by high-throughput sequencing. Soil physicochemical properties, fruit nutritional and functional components, antioxidant capacity, and electronic sensory traits were also evaluated. The application of LYB73 was associated with lower rhizosphere soil pH (5.52–6.82) and changes in several soil nutrient-related parameters. Microbial community analyses indicated that LYB73 treatment was accompanied by shifts in the composition of rhizosphere bacterial and fungal communities. For example, the relative abundance of Pseudomonadota increased in the JX treatment group, while Ascomycota was enriched in the JX and YPT treatment groups. At the genus level, taxa such as *Gemmatimonas*, *Saitozyma*, and *Cephalotrichum* showed increased relative abundance in some treated groups. Compared with the control, LYB73-treated fruits generally showed higher levels of reducing sugars, titratable acids, amino acids, total phenols, total flavonoids, and antioxidant capacity. The magnitude of these responses varied among cultivars: JX showed larger increases in total phenols, total flavonoids, and DPPH scavenging activity, WHJ showed a greater increase in amino acids and ABTS scavenging activity, and YPT showed the largest increase in superoxide anion scavenging activity. Electronic sensory analysis further suggested that LYB73 treatment affected taste and aroma-related traits, although the responses differed among cultivars. Correlation analysis showed that several dominant microbial genera were significantly associated with soil properties, fruit quality indices, and sensory indicators. Overall, these results suggest that pre-harvest application of *B. velezensis* LYB73 may influence rhizosphere microecology and fruit quality in a cultivar-dependent manner, providing preliminary support for its potential use in peach production.

## 1. Introduction

The peach (*Prunus persica* L.) is a perennial medium-sized tree of the Rosaceae family, native to China, and is regarded as an important economic crop [1,2]. Consumers highly favor peaches due to their beautiful fruit appearance, delicate flesh, unique flavor, and rich content of vitamins, amino acids, phenolic compounds, and various nutrients [3]. Longquanyi District in Chengdu, Sichuan Province, as a major production area for peaches in China, has nurtured several high-quality varieties such as “Jinxia,” “Wanhujing,” and “Youpantao” due to its unique climatic conditions and soil resources. With the improvement in living standards, consumers have placed higher demands on fruit quality, particularly emphasizing taste, nutritional value, and safety [4,5]. Aroma and taste play synergistic roles in shaping the overall flavor of food [6]. The formation of peach flavor is a dynamic process, and current research primarily focuses on changes in volatile organic compounds during fruit ripening. Peaches are abundant in diverse bioactive compounds and nutritional components, including vitamins, anthocyanins, flavonoids, phenolic compounds, and minerals [7,8]. Among these, phenolic and flavonoid compounds are plant secondary metabolites with prominent antioxidant activity, and they are widely distributed in various plant-based foods [9]. Their antioxidant properties are reflected in aspects such as free radical scavenging, metal chelation, and hydrogen donation [10]. However, there is limited research on the relationship between the sensory characteristics and nutritional composition of different peach varieties.

Peach quality is jointly regulated by various factors, including variety characteristics, soil conditions, climate change, maturity, and agricultural cultivation methods [11,12]. Among these, soil pH, nutrient availability, and microorganisms play a critical role in fruit development and quality formation [13]. Research indicates that soil pH has differential effects on tree growth and the absorption of certain elemental nutrients [14]. Additionally, the content of organic matter, nitrogen, phosphorus, and potassium in the soil is closely related to the growth and development of peach trees and fruit quality. Potassium is a crucial element in the growth and development of fruit trees, positively influencing photosynthetic characteristics and promoting the transport of photoassimilates [15], while also participating in the synthesis and transport of sugars and starches [16]. Sufficient nitrogen content improves the nutritional quality of the fruit, such as soluble sugars and vitamin C content [17], but excessive nitrogen supply can interfere with the absorption of other elements, reducing peach fruit quality [18]. Phosphorus accelerates plant development and enhances water-use efficiency [19]. Soil microbial communities (including bacteria, fungi, etc.), as vital components of the rhizosphere microenvironment, are also key facilitators of soil nutrient cycling. They not only participate in organic matter decomposition and nutrient mineralization [20] but also regulate plant nutrient uptake and growth through rhizosphere microbe–plant interactions [21]. However, current research on the impact of rhizosphere microorganisms on peach fruit quality remains relatively limited.

Long-term monoculture planting and excessive application of chemical fertilizers and pesticides may lead to degradation of soil physical and chemical properties, nutrient imbalance, and functional degradation of microbial communities in orchard soils, thereby affecting fruit quality and flavor [22,23]. In recent years, plant growth-promoting rhizobacteria (PGPR) have garnered widespread attention in research on improving crop quality and efficiency due to their environmentally friendly nature, long-lasting effects, and ability to enhance soil fertility [24,25]. PGPR can promote plant growth through various mechanisms such as secreting phytohormones, producing siderophores, solubilizing phosphorus and potassium, and chelating heavy metals, thereby improving crop tolerance to biotic and abiotic stresses and enhancing fruit quality [26,27]. *Bacillus velezensis* (*B. velezensis*) is recognized as a widely accepted plant growth-promoting rhizobacterium that can effectively improve fruit quality. Studies have found that *Bacillus velezensis* CE100 enhances strawberry fruit yield through dual mechanisms of direct biocontrol of phytopathogenic fungi and plant growth promotion [28]. It has been reported that *Bacillus velezensis* significantly reduces the occurrence of guava black spot disease by inhibiting the growth of the pathogen *Phyllosticta capitalensis*, disrupting cell membrane integrity, and increasing levels of malondialdehyde (MDA), hydrogen peroxide (H_2_O_2_), and superoxide anion (O^2−^), while reducing the activities of catalase (CAT) and peroxidase (POD), thereby effectively safeguarding fruit health [29]. Previous studies have confirmed that *B. velezensis* can effectively alleviate abiotic stress, improve the rhizosphere microecology, and enhance fruit yield and quality in grapes [30], strawberries [28], raspberries [31], tomatoes [32], and other fruits and vegetables [33,34,35]. However, systematic research on the pre-harvest application of *B. velezensis* in different varieties of peaches remains limited.

The application of microbial agents can have both positive and negative effects on plants. However, there is still a lack of detailed research on the effects of novel PGPR agents on different varieties of the same plant. Therefore, this study applied *Bacillus velezensis* LYB73 to the rhizosphere soil of three peach varieties (“Jinxia”, “Wanhujing”, and “Youpantao”). The main objectives of this study were to evaluate: (1) the effects of *Bacillus velezensis* LYB73 on the nutritional quality and antioxidant capacity of peach fruits across different varieties; (2) the impact of *Bacillus velezensis* LYB73 on the flavor characteristics and electronic sensory attributes of peaches, as well as the interrelationships between these attributes and fruit quality; (3) the regulatory effects of *Bacillus velezensis* LYB73 on the physicochemical properties of rhizosphere soil, microbial community diversity, and structural composition. This study provides preliminary evidence that LYB73 application is associated with changes in peach fruit quality, rhizosphere soil properties, and microbial community composition, providing a basis for future mechanistic studies.

## 2. Materials and Methods

### 2.1. Preparation of Bacterial Suspension

The strain *Bacillus velezensis* LYB73 used in this study was deposited at the China General Microbiological Culture Collection Center (CGMCC No. 34429). *Bacillus velezensis* LYB73 was inoculated into 100 mL of YD medium (sucrose 10 g/L, K_2_HPO_4_ 2 g/L, (NH_4_)_2_SO_4_ 1 g/L, MgSO_4_·7H_2_O 0.5 g/L, yeast extract 0.5 g/L, NaCl 0.1 g/L) for activation, and incubated in a shaker at 30 °C and 180 rpm for 12 h. It was then inoculated at 2% into fresh YD medium and cultured for 48 h. The culture was centrifuged at 8000 rpm for 10 min to remove the medium, followed by two washes with sterile water. The bacteria were then resuspended in sterile water to prepare a bacterial suspension, and the cell concentration was adjusted to approximately 1 × 10^8^ CFU/mL.

### 2.2. Location Description, Peach Plant Treatment, and Sampling

The field trial was conducted in 2025 at the Longquanyi District Changsong Peach Orchard (30°31′26.8536″ N, 104°16′51.6828″ E) in Chengdu City, Sichuan Province, China. Longquanyi District of Chengdu City is located in a subtropical humid climate zone with distinct four seasons and relatively abundant sunlight resources, with an annual average sunshine duration of 1645.6 h; favorable temperature conditions, with an annual average temperature of 16.5 °C and an average frost-free period of 289 days; ample rainfall, with an annual average precipitation of 957 mm, and the primary irrigation water source is groundwater. The basic physicochemical properties of the orchard soil are sandy loam (organic matter 20.2 g/kg, available nitrogen 95.3 mg/kg, available potassium 80.8 mg/kg, available phosphorus 140.6 mg/kg). This experiment was conducted on three peach varieties in the orchard: Jinxia (JX), Wanhujing (WHJ), and Youpantao (YPT). For each cultivar, uniform and healthy individual trees with similar growth status, tree age, and canopy size were selected and divided into the *Bacillus velezensis* LYB73 treatment group and the sterile-water control group, with three biological replicates in each group. The standard spacing between adjacent trees was 4 m. To prevent cross-contamination of rhizosphere microbial communities between treatment and control trees, a minimum separation distance of 8 m was maintained between any treatment tree and control tree within the same block (Figure S1). In the treatment group, 200 mL of the *Bacillus velezensis* LYB73 microbial suspension (1 × 10^8^ CFU/mL) was evenly applied to the soil surface within a 30 cm radius around each tree using a spray bottle, at full bloom stage and 30 days after flowering. The control group was treated with the same volume of sterile water to compare the effects of different treatments. At harvest, rhizosphere soil and fruit samples were collected from each of the three trees in both the treatment and control groups for all three cultivars. Rhizosphere soil samples were collected individually from each tree and processed separately. Three biological replicates were included for each variety-treatment combination (a total of 18 rhizosphere soil samples), and DNA extraction and sequencing were performed on each sample. Soil samples were taken from the root zone at a depth of 30 cm after removing the surface soil. Approximately 800 g of soil was then placed in sterile sealed bags using the quartering method to test soil physicochemical properties and microbial diversity. For fruit sampling, fifteen uniform, defect-free fruits were collected from each tree to ensure representativeness. Fruits from the same tree were mixed and homogenized into a single biological replicate. Each treatment–cultivar combination included three biological replicates, and each replicate was analyzed in triplicate for fruit quality and flavor determination.

### 2.3. Soil Element Content Detection

Soil pH was measured in a 1:2.5 suspension of soil to deionized water using the electrode method [36]. Soil organic matter (OM) content was determined by the potassium dichromate oxidation-external heating method [37]. Total nitrogen (TN) in soil was analyzed using the Kjeldahl method [38]. Total phosphorus (TP) and available phosphorus (AP) in soil were measured by the molybdenum-antimony anti-spectrophotometric method [39]. Total potassium (TK) and available potassium (AK) contents in soil were determined using the flame photometric method [40]. The multi-element concentrations in soil samples were detected by four-acid digestion-inductively coupled plasma spectrometry [41].

### 2.4. Soil DNA Extraction and Genomic Sequencing Analysis

Total genomic DNA was extracted from 0.25 g of fresh rhizosphere soil samples using a soil DNA kit (D5625-01, Omega Bio-tek, Norcross, GA, USA) following the manufacturer’s instructions. DNA was quantified using Qubit 2.0 (Invitrogen, Carlsbad, CA, USA). To assess bacterial and fungal communities, the V4 region of the bacterial 16S rRNA gene was amplified using primer pairs 515F (5′-GTGCCAGCMGCCGCGGTAA-3′) and 806R (5′-GGACTACHVGGGTWTCTAAT-3′), while the ITS1 region for fungi was amplified using ITS5-F: 5′-GGAAGTAAAAGTCGTAACAAGG-3′ and ITS2-R: 5′-GCTGCGTTCTTCATCGATGC-3′. All PCR mixtures contained 15 µL Phusion High-Fidelity PCR Master Mix, 0.2 µM primers, and 10 ng genomic DNA template. The PCR conditions included an initial denaturation at 98 °C for 30 s, followed by 30 cycles of denaturation at 98 °C for 10 s, annealing at 54 °C for 30 s, extension at 72 °C for 30 s, and a final extension at 72 °C for 5 min. PCR products were purified using AMPure XT Beads (Beckman Coulter Genomics, Danvers, MA, USA) and quantified with Qubit 2.0. Qualified PCR products were analyzed using the Agilent 2100 Bioanalyzer (Agilent Technologies, Palo Alto, CA, USA) and the Illumina library quantification kit (Kapa Biosciences, Woburn, MA, USA), then pooled and sequenced on the NovaSeq 6000 (PE250) platform. Sample data were demultiplexed from the sequencing output based on barcode sequences and PCR primer sequences. After removing the barcodes and primer sequences, FLASH (V1.2.11) [42] was used to assemble reads for each sample, generating Raw Tags. Cutadapt (v1.9) was then employed to match reverse primer sequences and trim remaining sequences. The assembled Raw Tags underwent stringent filtering using fastp software (V0.23.1) to obtain high-quality Clean Tags [43]. The tag sequences were compared against taxonomic annotation databases (Silva (138.1) for 16S, Unite (v9.0) for ITS) to detect chimeric sequences, which were subsequently removed to yield Effective Tags [44]. For the obtained Effective Tags, the DADA2 module in QIIME2 software (v202202) was used to derive final ASVs (Amplicon Sequence Variants) and feature tables [45].

### 2.5. Fruit Quality Analysis

The acid in the test solution was titrated with an alkaline solution, using phenolphthalein as the indicator to determine the titration endpoint. The titratable acid (TA) content [46] in the peach was calculated based on the consumption of the alkaline solution. Under alkaline conditions, the DNS reagent was used to determine the reducing sugar content in the peach. The amino acid (AA) content in the peach was calculated using the ninhydrin colorimetric method. The total phenol (FTP) content [47] in the fruit was measured using the Folin–Ciocalteu method. The total flavonoid content [48] in the peach was determined by the NaNO_2_^−^Al(NO_3_)_3_^−^NaOH colorimetric method. The DPPH (1,1-diphenyl-2-picrylhydrazyl) radical scavenging assay was performed according to the method described in the literature [49]. The ABTS (2,2′-azinobis(3-ethylbenzothiazoline-6-sulfonic acid ammonium salt)) radical scavenging capacity was determined based on the literature method [50]. The superoxide anion scavenging capacity was measured using the pyrogallol method [51].

### 2.6. Electronic Sensory Analysis

To objectively evaluate the flavor of different peach samples, electronic sensing technologies such as electronic nose and electronic tongue analysis were employed. The SA-402b taste analysis system (Intelligent Sensor Technology Co., Ltd., Atsugi, Japan) was used to measure taste values. This instrument consists of five sensors—sourness and bitterness (C00), astringency (AE1), sourness (CA0), saltiness (CT0), and umami (AAE)—and two standard electrodes. It can assess eight taste qualities of samples: sourness, bitterness, astringency, aftertaste-A (astringent aftertaste), aftertaste-B (sour and bitter aftertaste), umami, richness, and saltiness. Before the formal experiment began, the sensors were first cleaned in a cleaning solution for 90 s, rinsed twice with a reference solution for 120 s each, and zeroed at the equilibrium position for 30 s. The sample testing time was 30 s, outputting the initial taste value; the reference solution was rinsed for 3 s, and the sensor was inserted into a new reference solution to test the aftertaste for 30 s. After measurement, the average response values of each sensor were recorded for analysis. A portable E-nose system (PEN3, Win Muster Airsense Analytics Inc., Schwerin, Germany) was also used. This system is equipped with 10 sensors sensitive to various compounds: W1C (aromatic hydrocarbons), W5S (nitrogen oxides), W3C (ammonia, aromatic molecules), W6S (hydrides), W5C (olefins, aromatics, polar molecules), W1S (alkanes), W1W (sulfur compounds), W2S (alcohols, some aromatic compounds), W2W (aromatic hydrocarbons, sulfur-containing organic compounds), and W3S (alkanes and aliphatic compounds). For electronic nose analysis, 10 g of crushed sample pulp was transferred into a 50 mL headspace vial, with three vials per sample (technical triplicate). The vial was sealed with a screw cap equipped with a PTFE/silicone septum to ensure an airtight condition. All samples were incubated for 30 min for headspace enrichment before detection. During measurement, direct headspace aspiration was conducted by inserting the sampling needle into the sealed headspace vial to collect volatile odor compounds. The measurement conditions were set as follows: sampling time of 1 s; sensor self-cleaning time of 60 s; sample injection time of 5 s; injection flow rate of 400 mL/min; analysis sampling time of 80 s. After measurement, the average values were saved for analysis.

### 2.7. Statistical Analysis

All data were analyzed using IBM SPSS Statistics 27 (IBM, Armonk, NY, USA). Statistical analysis was performed using one-way analysis of variance (ANOVA), followed by post hoc Tukey tests to evaluate the effects of different treatments on fruit quality and soil physicochemical properties. In this study, a *p*-value less than 0.05 was considered statistically significant. Means and standard deviations were calculated using Excel 2021 (Microsoft, Redmond, WA, USA). Radar charts, bar charts, and box plots were generated using Origin 2021 software (OriginLab, Northampton, MA, USA). Alpha-diversity indices, including Chao1, Shannon, Simpson, and Pielou’s evenness, were calculated using the vegan package (v2.6.2) in RStudio (v2024.09.1). Beta-diversity analysis based on weighted UniFrac distance was performed in QIIME2 to compare microbial community composition among samples. Principal coordinate analysis (PCoA) was conducted and visualized using the ade4 package (v1.7.15) and ggplot2 package (v3.3.6) in RStudio. Additionally, the Linear Discriminant Analysis Effect Size (LEfSe) method with a LDA threshold of >4 and the Kruskal–Wallis test (*p* < 0.05) were used to identify significantly different taxonomic at the genus level [52]. Spearman correlation analysis was conducted using the Genescloud web platform (https://www.genescloud.cn/home, accessed on 31 March 2026).

## 3. Results and Discussion

### 3.1. Evaluation of Peach Fruit Quality

**Figure 1:**
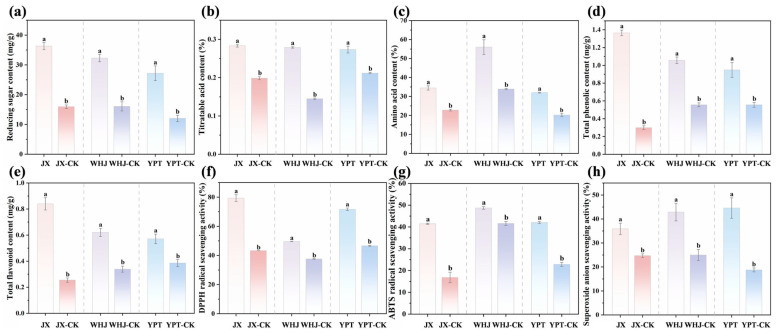
Effects of *Bacillus velezensis* LYB73 application on the fruit quality of different peach varieties. (**a**) Reducing sugar content; (**b**) titratable acid content; (**c**) amino acid content; (**d**) total phenolic content; (**e**) total flavonoid content; (**f**) DPPH radical scavenging activity; (**g**) ABTS radical scavenging activity; (**h**) superoxide anion scavenging activity. Error bars indicate standard deviation. Different letters above the bars indicate significant differences between the bacterial agent-treated and control groups for each variety (based on ANOVA, *p* < 0.05).

Compare the changes in fruit nutritional quality, functional components, and antioxidant capacity of three peach varieties (JX, WHJ, YPT) under *Bacillus velezensis* LYB73 treatment and control (CK) (Figure 1). Soluble sugars, titratable acids, and amino acid content are the main substances determining fruit quality and nutritional value [53]. The results showed that the contents of reducing sugar, titratable acidity, and amino acids in all treatment groups were significantly higher than those in the control group (*p* < 0.05). Among them, the JX treatment group exhibited the largest increase in reducing sugar relative to the JX-CK group, while the WHJ treatment group showed the greatest enhancement in titratable acidity and amino acid contents compared with the WHJ-CK group. The contents and proportional composition of sugars and organic acids are key factors affecting the flavor perception of peach fruit [54]. The sugar–acid ratio of fruit in all treatment groups was higher than that in the corresponding control groups, indicating that the increase in acidity did not exert an adverse effect on the overall flavor balance. The balanced ratio of sugar to acid observed in this study may be associated with the regulatory effect of LYB73 on enzyme activities in plants [55,56]. Amino acids, as fundamental components of proteins, are crucial for human nutrition and also play a key role in determining the flavor characteristics and nutritional quality of fruits [57]. Amino acid metabolism is one of the most important biochemical adaptations to many environmental stresses, such as drought, low temperature, and high temperature before and after harvest [58]. The bacterial treatment significantly increased the content of total phenols and total flavonoids, which are functional components, in the fruits of all three varieties (*p* < 0.05). The JX variety was the dominant performer, with a total phenol content in the JX group being 4.53 times that of the JX-CK group, far exceeding the increases in WHJ (1.90 times) and YPT (1.71 times). The total flavonoids in the JX group were 3.29 times those of the JX-CK group, while the WHJ- and YPT-treated groups showed 1.83 times and 1.48 times increases compared to their respective CK groups. *Bacillus velezensis* LYB73 treatment significantly enhanced the scavenging capacity of ABTS, DPPH, and superoxide anions in the fruits of all three varieties. The study found that in fruits treated with the microbial agent, there was a significant positive correlation between the accumulation of phenolic and flavonoid compounds and antioxidant capacity, consistent with previous findings in strawberries [59] and tomatoes [60]. The WHJ group had the highest ABTS scavenging rate (48.74%), the JX variety showed the highest DPPH scavenging rate (79.35%), which was 1.83 times that of the CK group, and the YPT variety exhibited the most significant increase in superoxide anion scavenging rate, being 2.37 times that of the YPT-CK group. Overall, the JX variety performed best in DPPH scavenging rate and total phenol/flavonoid accumulation, the WHJ variety showed significant advantages in ABTS scavenging rate and amino acid accumulation, and the YPT variety excelled in superoxide anion scavenging rate. The bacterial treatment did not alter the inherent antioxidant characteristics of the varieties but achieved significant enhancements based on their original traits.

### 3.2. Electronic Sensory Evaluation of Peach Fruit

**Figure 2:**
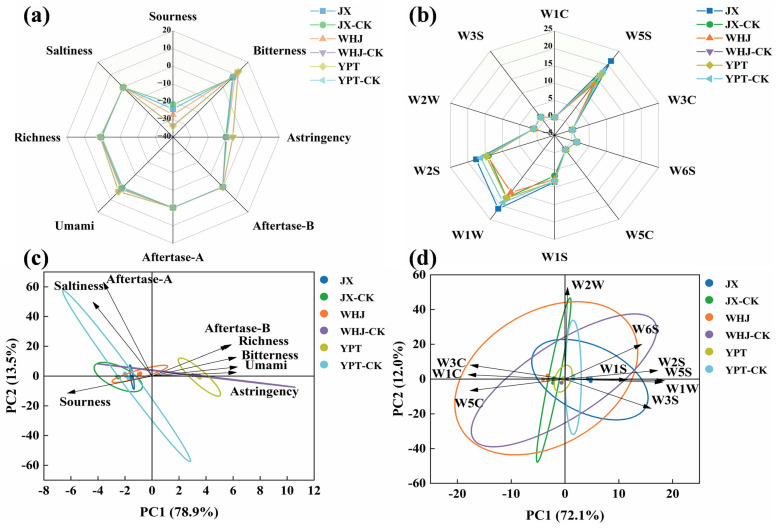
Effects of *Bacillus velezensis* LYB73 application on the flavor characteristics of different peach varieties. (**a**) Flavor radar plots of peach fruits from different varieties generated by an electronic tongue; (**b**) Flavor radar plots of peach fruits from different varieties generated by an electronic nose; (**c**) Principal component analysis (PCA) of taste in peach fruits from different varieties; (**d**) Principal component analysis (PCA) of aroma in peach fruits from different varieties.

**Figure 3:**
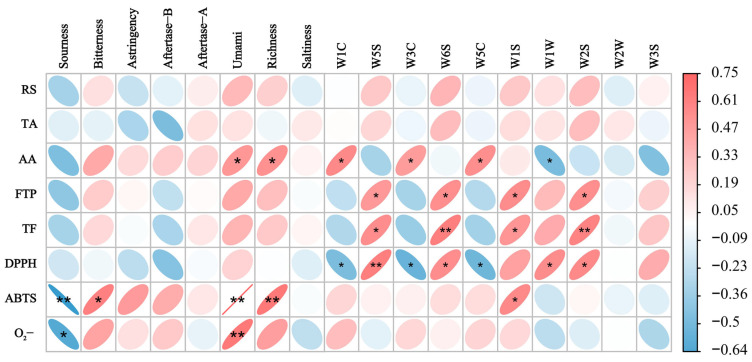
Analysis of the correlation between peach fruit quality and electronic sensory indicators; color gradients indicate correlation strength (red: positive, blue: negative; ** *p* < 0.01, * *p* < 0.05). Only statistically significant correlations (*p* < 0.05) are shown.

The electronic tongue can use electronic sensors to detect taste characteristics and mimic human gustatory perception [61]. Analysis of the radar chart (Figure 2a) and bar chart (Figure S2a) of the electronic tongue responses showed that the JX and YPT treatment groups had higher absolute values for sourness, lower absolute values for astringency, and significantly higher bitterness compared to the JX-CK and YPT-CK groups (*p* < 0.05), while Umami and Richness were also significantly enhanced (*p* < 0.05). Conversely, the WHJ-CK group had a higher absolute value for sourness and a lower absolute value for astringency than the WHJ group. The bitterness, Umami, and Richness of WHJ were significantly lower than those of WHJ-CK (*p* < 0.05). Bitterness and astringency are closely related to the taste quality of the fruit. Bitterness is determined by the composition and ratio of compounds such as flavonoids, phenolic acids, terpenes, and alkaloids [62], and phenolic acids are often associated with astringency and bitterness of fruits [63]. Changes in bitter and astringent taste attributes may have been associated with variations in the content of flavonoids and phenolic acids. Using sensor response values as input variables, PCA was performed on the electronic tongue dataset (Figure 2c) to distinguish between peach varieties treated with or without microbial agents. PC1 explained 78.9%, and PC2 explained 13.5% of the total variation. WHJ and WHJ-CK were clearly separated, while the other two varieties showed less distinct separation. The electronic nose can sensitively capture odor information from samples, as minor changes in volatile compounds may lead to differences in sensor responses. Based on electronic nose data, the radar chart (Figure 2b) and bar chart (Figure S2b) revealed that, compared to the other two varieties, the JX group had significantly higher values for W1S, W2S, W3S, and WIW sensors, suggesting stronger sensor responses associated with alcohols, aliphatic compounds, and sulfur-containing compounds compared to JX-CK. The WHJ treatment group showed significantly higher sensor response values for W1C, W3C, and W5C than the WHJ-CK group (*p* < 0.05). These sensors are sensitive to aromatic compounds, ammonia, and alkenes. However, most electronic nose sensor response values showed no significant difference between YPT and YPT-CK, indicating that their aroma profiles and the content of aroma-related compounds were largely similar. PCA was used to describe sample relationships (Figure 2d), with PC1 and PC2 accounting for 72.1% and 12.0% of the total variance, respectively, cumulatively explaining 84.1%. WHJ was positioned farthest to the right on PC1, while JX was farthest to the left, indicating changes in flavor characteristics. Additionally, all WC electronic nose sensors, including W1C, W3C, and W5C, are clustered on the left side, reflecting the association between these sensors and samples grouped on the left. All WS electronic nose sensors, including W1S, W2S, W3S, W5S, W6S, and W1W, are also clustered on the right side, reflecting their association with samples grouped on the right. Correlation analysis between fruit quality and electronic sensory data (Figure 3) showed that, in terms of taste, Umami and Richness were positively correlated with amino acids (*p* < 0.05) and with ABTS scavenging capacity (*p* < 0.05). For aroma indicators, amino acids were positively correlated with the response values of WC (W1C, W3C, and W5C) sensors (*p* < 0.05), suggesting that amino acids may be positively associated with the responses of sensors related to aromatic compounds, ammonia, and alkenes. Meanwhile, total flavonoids and total phenols were positively correlated with the response values of WS (W1S, W2S, W5S, W6S) sensors (*p* < 0.05), indicating that flavonoids and phenols may influence the synthesis of alkanes, alcohols, hydrides, and nitrogen oxides in peaches.

### 3.3. Soil Microbial Diversity Analysis

**Figure 4:**
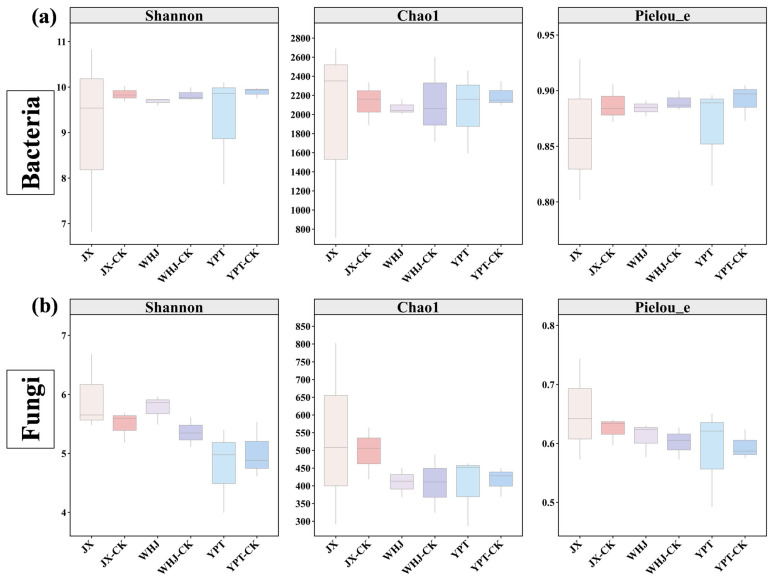
Effects of *Bacillus velezensis* LYB73 Application on α-Diversity of Soil Microorganisms in Different Peach Root Zones (**a**) α-diversity levels in the bacterial community; (**b**) α-diversity levels in the fungal community.

The Shannon index, Chao1 index, and Pielou_e evenness index were used to evaluate the α-diversity of soil bacterial and fungal communities in different varieties of peaches after bacterial agent treatment (Figure 4). The application of bacterial agents to different varieties resulted in differences in the Shannon index and Chao1 index values of bacterial communities, but most differences were not significant. The Pielou_e evenness of bacteria in JX, WHJ, and YPT decreased compared to JX-CK, WHJ-CK, and YPT-CK, respectively, which may reflect the increased dominance of specific taxa after LYB73 application. Among the fungal communities, the JX treatment group exhibited the highest mean Shannon index. The cultivar-specific responses of fungal alpha diversity may be closely associated with differences in root exudate composition among cultivars [64]. The Chao1 index and Pielou_e evenness showed little variation among the three varieties. Changes in plant-associated microbial community diversity are a typical effect of microbial agent application and have been analyzed in studies of other crops (e.g., strawberries, tomatoes, raspberries) [31,59,65]. The bacterial agents treatment was accompanied by shifts in rhizosphere microbial community composition, with taxa adapted to the altered microenvironment becoming more abundant, while the variety-specific rhizosphere microenvironments shaped unique indigenous microbial communities, potentially leading to varietal differences in microbial responses to the agents [66].

Principal coordinate analysis (PCoA) plots revealed the β-diversity variations in bacterial and fungal communities between JX, WHJ, and YPT bacterial agent treatments and the control group in the samples (Figure S3). The rhizosphere soil bacterial communities of the three varieties of peach treated with bacterial agents showed clear separation compared to the control group. In contrast, the β-diversity of fungal communities exhibited greater differences than bacterial communities, regardless of bacterial agent application or variety. Consistent with previous reports, *W. anomalus* had a significantly greater impact on fungal communities than on bacterial communities when applied to kiwifruit [67], and *D. hansenii* bacterial treatment on strawberries influenced fungal microbiota structure more than bacterial communities [68]. The variability in fungal communities and the relative stability of bacterial communities highlight the dynamic nature of the soil microbiome and its potential effects on peaches. Bacterial community structure showed smaller differences between treatment and control groups compared to fungal communities. This may be due to differences in the ecological niches occupied by bacteria and fungi or the microbial interactions of *Bacillus velezensis* LYB73, which primarily target fungal taxa [69,70]. Higher fungal diversity has been associated with enhanced disease suppression in previous studies, thereby enhancing fruit health [71].

### 3.4. Soil Microbial Community Composition and Differences

**Figure 5:**
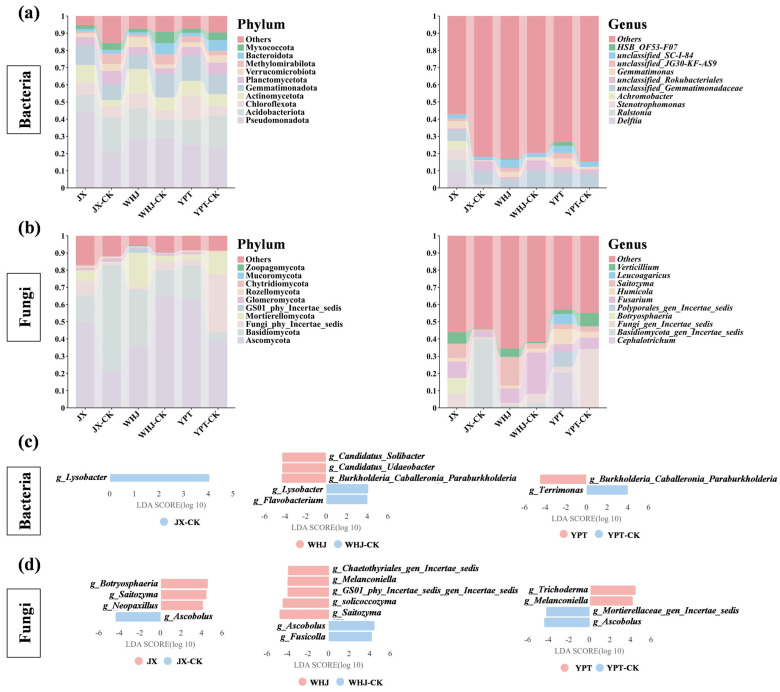
Effects of *Bacillus velezensis* LYB73 Application on the Rhizosphere Microbial Communities of Different Peach Varieties. (**a**) Stacked bar charts of bacterial phylum and genus abundances; (**b**) Stacked bar charts of fungal phylum and genus abundances; (**c**) Bar charts of Linear Discriminant Analysis (LDA) Effect Size (LEfSe) scores for bacterial genus differences; (**d**) Bar charts of Linear Discriminant Analysis (LDA) Effect Size (LEfSe) scores for fungal genus differences.

We analyzed the top 10 phyla and 10 genera of bacterial (Figure 5a) and fungal (Figure 5b) relative abundance. The results showed that the *Bacillus velezensis* LYB73 treatment altered the relative abundance of bacterial phyla in each variety, with the trends of change being variety-specific. The most abundant bacterial phyla in the soil were identified as Pseudomonadota, Acidobacteriota, Chloroflexota, Actinomycetota, and Gemmatimonadota. Pseudomonadota includes numerous taxa associated with plant hormone regulation, soil health restoration, and crop resilience enhancement [72,73]. The relative abundance of Pseudomonadota was higher in the JX and YPT treatment groups than in their corresponding controls, whereas it slightly decreased in WHJ. Chloroflexota and Actinomycetota are typically relatively abundant in farmland soils and may contribute to the connectivity of soil microbial community network nodes and plant health [74,75]. Chloroflexota exhibited the highest relative abundance in the YPT treatment group at 13.90%, and Actinomycetota showed the highest relative abundance in the WHJ treatment group at 14.12%. In contrast, the relative abundances of Planctomycetota, Bacteroidota, and Myxococcota in the treatment groups of all three peach cultivars were slightly lower than those in their respective control groups. Among the top 10 bacterial genera, JX showed substantial increases in the relative abundances of *Delftia* (9.22% vs. 0.59%), *Ralstonia* (7.26% vs. 0.92%), *Stenotrophomonas* (5.93% vs. 0.29%), and *Achromobacter* (4.95% vs. 0.56%) compared to JX-CK. Although the relative abundances of *Ralstonia* and *Stenotrophomonas* in the WHJ and YPT treatment groups increased compared to WHJ-CK and YPT-CK, their proportions remained low. *Gemmatimonas* exhibited increased relative abundance in the treatment groups of all three varieties (JX, YPT, and WHJ), becoming their dominant genus. These genera have been reported to be associated with plant growth promotion and soil health improvement in previous studies. For example, *Delftia* [76], *Ralstonia* [77], *Stenotrophomonas* [78], and *Achromobacter* [79] can tolerate heavy metals in soil and demonstrate good capabilities in heavy metal bioabsorption; *Gemmatimonas* [80] can effectively increase soil nitrogen content and mutually enhance soil urease and protease activities. Notably, the genus *Ralstonia* also includes the notorious soilborne pathogen *Ralstonia solanacearum*, which causes destructive bacterial wilt in hundreds of plant species, including economically important crops such as tomato, potato, banana, eggplant, and pepper [81]. Although no obvious disease symptoms were observed in peach trees during the present field investigation, the enrichment of these potentially pathogenic genera reminds us that long-term field monitoring and further verification of pathogenicity are still required to clarify the potential ecological risks posed by LYB73-induced shifts in the rhizosphere microbial community.

At the phylum level, the fungal community was dominated by Ascomycota, Basidiomycota, and Mortierellomycota. The abundance of Ascomycota in the JX (49.75%) and YPT (62.60%) treatment groups was significantly higher than that in the corresponding JX-CK and YPT-CK groups (*p* < 0.05), while the Basidiomycota showed the opposite trend. In JX-CK, the relative abundance of this phylum reached 61.55%, far exceeding the 15.59% in the JX treatment group. Ascomycota is the largest phylum of fungi, encompassing numerous saprophytic and symbiotic species, and its increased abundance may be related to organic matter decomposition and nutrient release [82]. Many taxa within Basidiomycota are involved in lignin degradation, and changes in their abundance may reflect treatment-induced alterations in the composition of rhizosphere organic matter [83]. Basidiomycota and Mortierellomycota were the characteristic dominant phyla in the WHJ treatment group, with relative abundances of 32.91% and 19.92%, respectively. Studies indicate that Mortierellomycota is closely associated with soil nutrients, possesses phosphorus-solubilizing capabilities, and can produce plant hormones, benefiting plant growth [84,85]. At the genus level, *Fusarium* had a higher relative abundance of 9.49% in the JX treatment group compared to 3.42% in the JX-CK group. *Saitozyma* was enriched in the WHJ treatment group, while *Cephalotrichum* and *Humicola* were the core dominant genera in the YPT treatment group. The genus *Fusarium* is taxonomically large and ecologically diverse, encompassing saprophytic, endophytic, and pathogenic species that infect a wide range of edible fungi and plant hosts [86,87]. Furthermore, *Fusarium* represents one of the most important pathogens of cereal and other crops, particularly wheat, maize, and soybean, imposing substantial constraints on modern agricultural production [88,89]. Therefore, the enrichment of *Fusarium* under LYB73 treatment cannot be uncritically regarded as beneficial and should be carefully evaluated from an ecological safety perspective. The abundance of *Saitozyma* correlates with soil organic matter (OM) and available phosphorus (AP), suggesting its role in nutrient mobilization. *Cephalotrichum* is a common soil saprophyte that may participate in the decomposition of organic matter [90].

Combined with LEfSe (LDA threshold 4.0) detection, further identification of microbial differences between the treatment and control groups in soil samples of different peach varieties after microbial agent treatment was conducted at the genus level (Figure 5c,d). The results showed that the JX treatment group was enriched with fungal genera such as *Botryosphaeria* and *Fusarium*, while the WHJ treatment group was enriched with bacterial genera *Candidatus_Udaeobacter* and *Candidatus_Solibacter*, and fungal genera *Saitozyma* and *Solicoccozyma.* The YPT microbial agent treatment group was enriched with fungal genera *Trichoderma* and *Melanconiella*. These taxa can serve as indicator taxa responsive to *Bacillus velezensis* LYB73 treatment, and their functions require further study. Imbalance in the soil microbial community of the fruit may lead to changes in fruit nutritional quality and is also a significant factor in fruit disease occurrence [91]. The composition and function of soil microbial communities are influenced by a combination of factors, including plant genotype, environmental conditions, agricultural management practices, plant developmental stages, and interactions among microorganisms [66,92]. These factors collectively determine the composition of the rhizosphere soil microbial community, thereby affecting fruit quality [93]. The prevalence of these dominant microbial taxa may be attributed to the specific growth environment of peaches, unique fruit characteristics, microbial agent interactions, and soil microbial interactions. However, as pre-treatment soil samples were not collected, the possibility that some of the observed differences in microbial community composition reflect pre-existing variation among trees cannot be ruled out.

### 3.5. Soil Physicochemical Analysis

**Figure 6:**
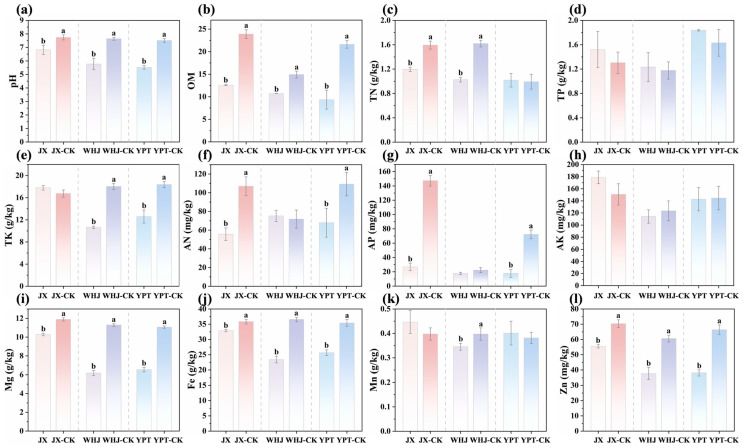
Effects of *Bacillus velezensis* LYB73 application on physicochemical properties of soil in the root zone of different peach varieties. (**a**) pH; (**b**) organic matter; (**c**) total nitrogen; (**d**) total phosphorus; (**e**) total potassium; (**f**) available nitrogen; (**g**) available phosphorus; (**h**) Available potassium; (**i**) Mg; (**j**) Fe; (**k**) Mn; (**l**) Zn. Error bars indicate standard deviation. Different letters above the bars indicate significant differences (*p* < 0.05) between each variety’s bacterial inoculant treatment and the control.

Soil physicochemical parameters are another key indicator of soil quality. Pre-harvest *Bacillus velezensis* LYB73 treatment significantly altered the physicochemical properties of rhizosphere soil across three peach cultivars (Figure 6). Regarding soil pH, the mean values in the treatment groups of all three cultivars were significantly lower than those in the sterile water control groups (*p* < 0.05), decreasing to 6.82, 5.77, and 5.52 for JX, WHJ, and YPT, respectively. The lower rhizosphere pH observed in the treatment group may be associated with the potential production of organic acids by LYB73 [94]. Additionally, this strain may promote nitrification, as the nitrification of ammonium nitrogen releases H^+^, leading to soil acidification [95]. A pH drop to the weakly acidic range (5.5–7) enhances the availability of nutrients such as iron, manganese, zinc, and copper, which may indirectly facilitate plant uptake of these elements [96]. Compared with the control groups, the soil organic matter (OM) content was lower in the LYB73-treated groups, with average values of 12.59, 10.72, and 9.37 g/kg for JX, WHJ, and YPT cultivars, respectively. The lower OM content in LYB73-treated soils may reflect a combination of microbial activity, plant uptake, spatial heterogeneity, and/or pre-existing differences among trees [97,98]. Because pre-treatment soil samples were not collected, we cannot determine whether this decrease was caused by LYB73 treatment. Long-term, multi-season monitoring is required to evaluate whether LYB73 application leads to sustained changes in soil organic matter. Soil pH and OM content interact to regulate crop nutrient availability through soil phosphatase activity [99]. Soil pH and OM content are critical parameters that influence soil quality, and maintaining them within optimal ranges is vital for soil health and for guiding cultivation strategies. Regarding nitrogen, phosphorus, and potassium, total nitrogen (TN) and total potassium (TK) contents were generally lower in the treatment groups than in the controls, while total phosphorus (TP) showed no significant difference. Available nitrogen (AN) and available phosphorus (AP) exhibited cultivar-specific responses, with both being significantly lower in the JX and YPT treatment groups compared to their respective controls, whereas no significant differences were observed between the WHJ treatment group and its control. The changes in total and available nutrients showed cultivar-specific patterns. These patterns may reflect the combined effects of microbial activity, plant nutrient uptake, and pre-existing soil heterogeneity, but the underlying processes require further validation. Notably, the significant increase in available potassium in the JX treatment group provided critical mineral nutrition for the high accumulation of reducing sugars and functional components in the fruit of this cultivar [15,100]. The trends for magnesium (Mg), iron (Fe), and zinc (Zn) were similar. Mg content in the JX treatment group was 13.56% lower than in JX-CK; in WHJ, it was 45.31% lower than in WHJ-CK; and in YPT, it was 40.90% lower than in YPT-CK. Magnesium, a core component of chlorophyll, is involved in photosynthesis and enzyme activation [101]. Fe content in the JX, WHJ, and YPT treatment groups decreased by 8.15% to 35.97% compared to their respective controls. Zn content in the treatment groups also decreased by 14.82 to 28.25 mg/kg relative to the controls. The decline in trace elements (Fe, Mn, Zn) is particularly noteworthy. Iron, manganese, and zinc are vital nutrients for organisms across all life domains, participating in cellular metabolism, acting as cofactors for various enzymes, and forming biological complexes with metals that perform critical functions, including defense against oxidative stress [102,103]. Whether these decreases reflect enhanced plant uptake or other soil processes remains to be determined.

### 3.6. Correlation Between Soil Microbial Communities and Fruit Quality, Electronic Sensory Evaluation of Fruits, and Soil Physicochemical Properties

**Figure 7:**
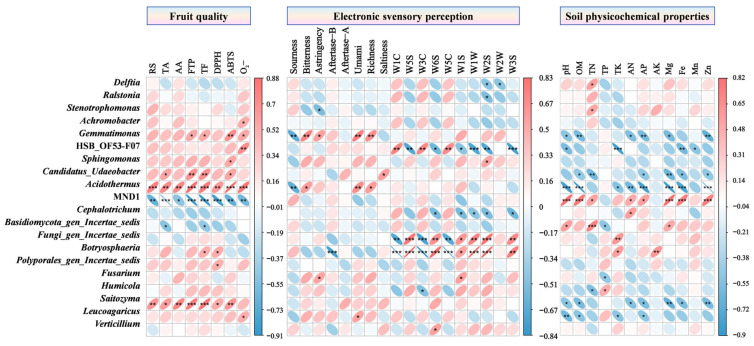
Spearman’s correlation analysis of peach fruit quality, electronic sensory evaluation, soil physicochemical properties, and dominant genera. The color gradient indicates correlation strength (red: positive; blue: negative; *** *p* < 0.001, ** *p* < 0.01, * *p* < 0.05). Only statistically significant correlations (*p* < 0.05) are shown. Abbreviations: RS, reducing sugars; TA, titratable acids; AA, amino acids; FTP, total phenolic content; TF, total flavonoids; O_2_^−^, superoxide anion; OM, organic matter; TN, total nitrogen; TP, total phosphorus; TK, total potassium; AN, available nitrogen; AP, available phosphorus; AK, available potassium.

To deeply analyze the intrinsic relationships between rhizosphere microbial community changes and fruit quality, flavor characteristics, and soil physicochemical properties, this study conducted Spearman correlation analysis (Figure 7) between the top 10 dominant bacterial and fungal genera in peach rhizosphere soil and various environmental factors. The heatmap results revealed significant correlations (|r| > 0.6, *p* < 0.05) between microbial taxa and multiple fruit quality indicators, revealing potential associations between *Bacillus velezensis* LYB73 treatment, rhizosphere microbial community composition, fruit quality, and soil traits. Among the dominant bacterial genera, *Gemmatimonas*, *Candidatus_Udaeobacter*, and *Acidothermus* showed significant positive correlations with multiple fruit quality indicators (*p* < 0.05). *Gemmatimonas* exhibited significant positive correlations with total phenols, total flavonoids, ABTS radical scavenging capacity, and superoxide anion scavenging capacity (*p* < 0.05). *Candidatus_Udaeobacter* showed significant positive correlations with titratable acidity, total phenols, total flavonoids, and ABTS radical scavenging capacity (*p* < 0.05). *Acidothermus* was significantly positively correlated with basic nutritional components, total phenols, total flavonoids, and antioxidant activity (*P* < 0.05). The genus MND1 exhibited a negative correlation trend with multiple quality indicators. This genus was more abundant in the control groups of the three varieties and showed relatively smaller improvements in fruit quality, possibly due to its negative contribution. Among the dominant fungal genera, *Botryosphaeria* was positively correlated with total flavonoids and DPPH radical scavenging capacity. The abundance of *Saitozyma* showed significant positive correlations with reducing sugars, titratable acidity, amino acids, total phenols, total flavonoids, and antioxidant indices (DPPH and ABTS scavenging rates) (*p* < 0.05).

In the electronic tongue indicators, *Gemmatimonas* and *Acidothermus* showed significant positive correlations with Umami, Richness, and Bitterness, and significant negative correlations with Sourness (*p* < 0.05). This indicates that microbial taxa showed divergent correlational patterns with different taste qualities. In the electronic nose indicators, the response values of sensors W1C, W3C, and W5C were significantly positively correlated with HSB_OF53-F07, while W5S, W6S, W1S, W1W, W2S, and W3S showed significant positive correlations with *Fungi_gen_Incertae_sedis* and *Botryosphaeria* (*p* < 0.05). These results suggest that specific bacterial and fungal taxa showed correlations with distinct classes of volatile compounds, raising the hypothesis that they may contribute to variety-specific aromatic profiles through mechanisms that require further investigation.

Soil pH showed significant negative correlations with several microbial genera (*p* < 0.05), including *Gemmatimonas,* HSB_OF53-F07, *Acidothermus*, *Saitozyma*, and *Leucoagaricus*. These results suggest that the higher relative abundance of these taxa may be associated with the lower rhizosphere pH observed under LYB73 treatment. One possible explanation is that microbial metabolic activities, including the production of organic acids, may contribute to rhizosphere acidification; however, this hypothesis requires further experimental validation. Organic matter (OM) content was negatively correlated with most bacterial genera, with particularly significant negative correlations observed for *Gemmatimonas* and *Acidothermus* (*p* < 0.01). Available nutrients (AN, AP, AK) showed weak and mostly negative correlations with microbial genera, possibly because nutrients are rapidly absorbed by plants, reducing residual levels in the soil. For metal elements, Mg, Fe, and Zn exhibited significant positive correlations with MND1 but significant negative correlations with other abundant microorganisms (*p* < 0.05), such as *Candidatus_Udaeobacter*, *Acidothermus*, and *Saitozyma*. This could be attributed to the *Bacillus velezensis* LYB73 treatment enriching specific microbial taxa, promoting the activation of trace elements in the soil and their transport to plants, thereby reducing the content of these elements in the soil reservoir. The Spearman correlation analysis and heatmap results suggested that genera such as *Gemmatimonas* and *Acidothermus* were closely associated with fruit quality and soil properties. These results suggest that shifts in the rhizosphere microbiome are associated with the observed changes in soil and fruit characteristics.

## 4. Conclusions

In this study, pre-harvest application of *Bacillus velezensis* LYB73 to three peach cultivars improved fruit quality and modified fruit aroma and taste attributes. In addition, significant changes were observed in soil physicochemical properties and bacterial and fungal communities. These changes included cultivar-specific shifts in soil nutrients, divergent soil element profiles, altered microbial diversity, and restructured bacterial and fungal community composition. Correlation analysis revealed significant associations between several dominant microbial genera, soil physicochemical parameters, and fruit quality traits, suggesting that shifts in the rhizosphere microbiome may be related to the observed changes in soil and fruit characteristics. Enrichment of genera containing potentially pathogenic members, such as *Ralstonia* and *Fusarium*, highlights the need for long-term monitoring. Collectively, these findings provide preliminary insights into the effects of LYB73 on fruit and soil health across different peach cultivars. Future studies with larger sample sizes and multi-year field monitoring are warranted to validate these observations.

## Data Availability

The original contributions presented in this study are included in the article/Supplementary Material. Further inquiries can be directed to the corresponding authors.

## Supplementary Materials

The following supporting information can be downloaded at: https://www.mdpi.com/article/10.3390/foods15111852/s1, Figure S1: Field arrangement diagram; Figure S2: Effects of *Bacillus velezensis* LYB73 application on the aroma and texture of different peach varieties; Figure S3: Effects of *Bacillus velezensis* LYB73 application on β-diversity of soil microorganisms in the rhizosphere of different peach trees.
