# Supplementary material for: The Influence of Pre-Harvest Application of Bacillus velezensis LYB73 on the Rhizosphere Soil Properties, Microbial Communities, Fruit Quality, and Flavor Characteristics of Different Varieties of Peaches
Source: Foods. 2026 May 23;15(11):1852. doi: 10.3390/foods15111852 (PMC13256861; doi:10.3390/foods15111852)
Supplement: Supplementary file 1 [file foods-15-01852-s001.zip › foods-4286538-supplementary.pdf]

Supplementary materials

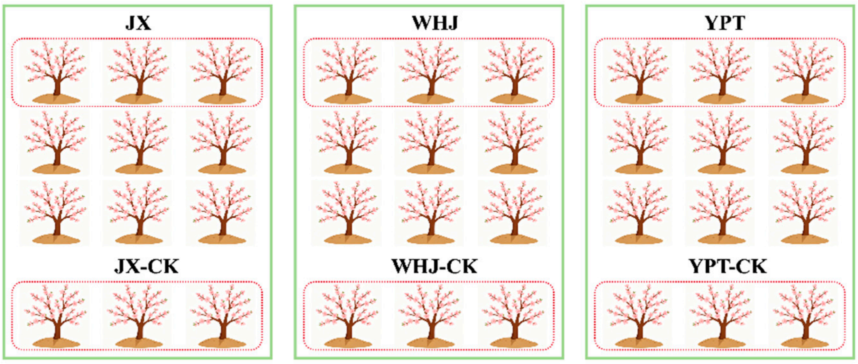

Figure S1 Field arrangement diagram.

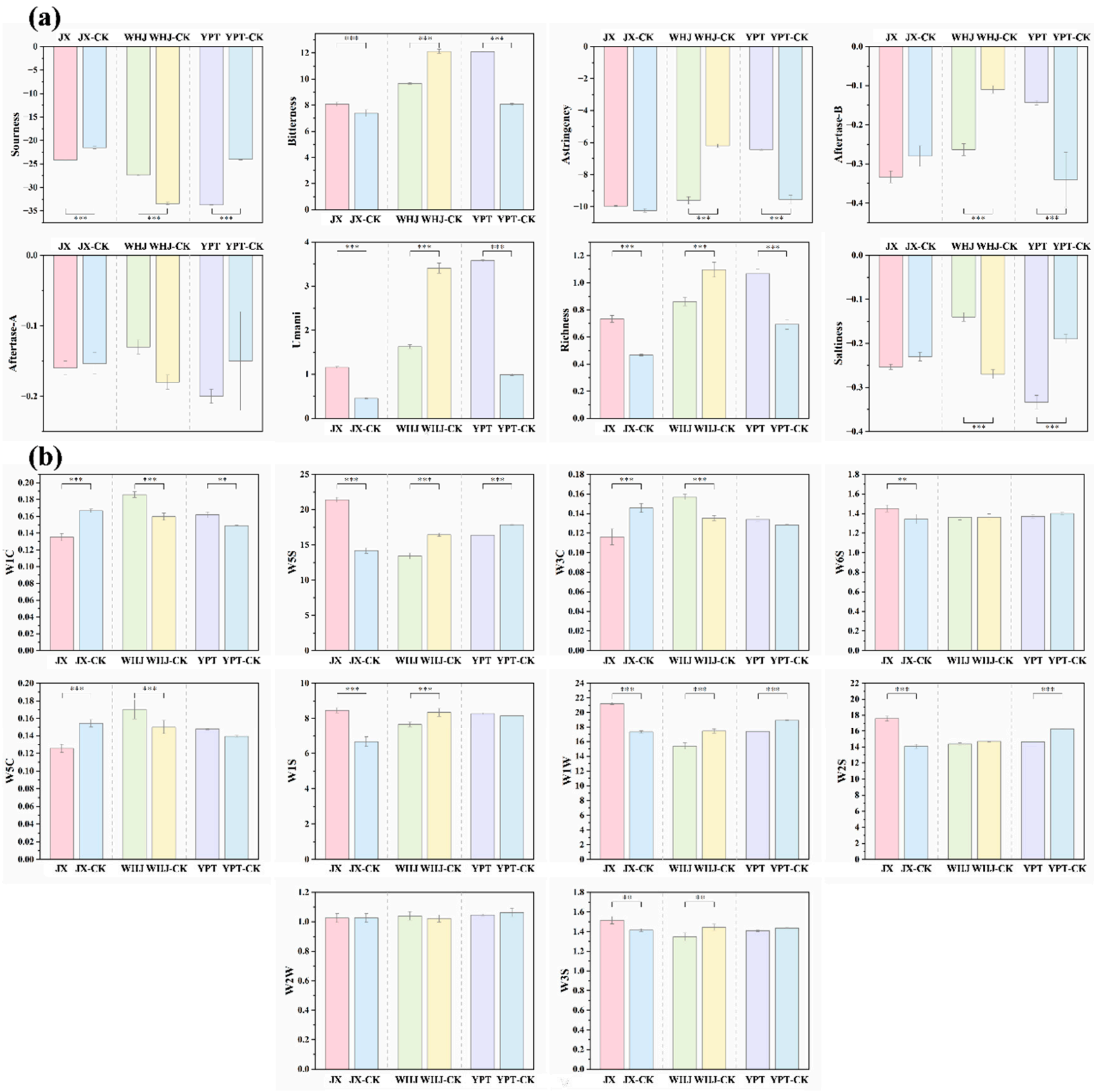

Figure S2 Effects of *Bacillus velezensis* LYB73 application on the aroma (a) and texture (b) of different peach varieties. Error bars indicate standard deviation. Significant differences are indicated by *** ($P < 0.001$), ** ($P < 0.01$), and * ($P < 0.05$).

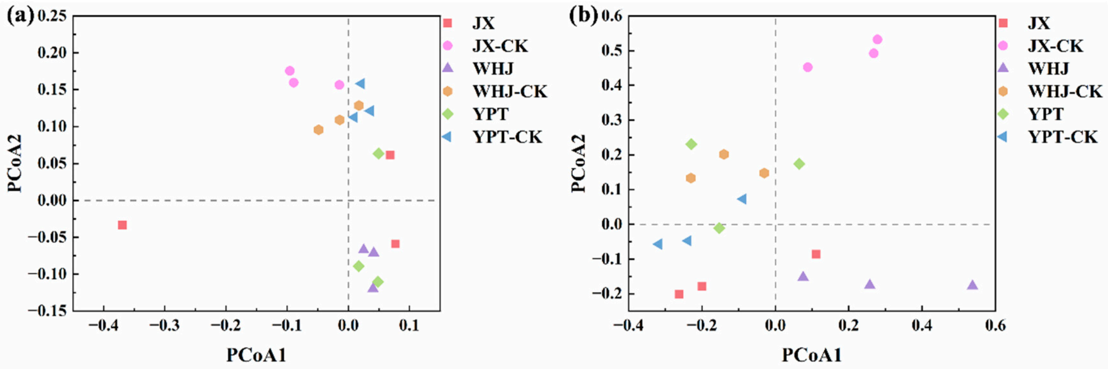

Figure S3 Effects of *Bacillus velezensis* LYB73 application on β-diversity of soil microorganisms in the rhizosphere of different peach trees: (a) bacteria; (b) fungi.
